# Exploratory survey study of differences in knowledge, attitudes, and practices of outpatient older adult clinical care between dermatologists, primary care physicians and geriatricians across three academic medical centers

**DOI:** 10.1186/s12877-025-06628-8

**Published:** 2026-01-29

**Authors:** Xiaochen Zhong, Twan Sia, Warren M. Perry, Shufeng Li, Caroline Park, Silvia Tee, Deborah M. Kado, Howa Yeung, Katrina Abuabara, Anne Lynn S. Chang

**Affiliations:** 1 Department of Dermatology, University of California San Francisco School of Medicine, San Francisco, USA; 2 Department of Dermatology, Stanford University School of Medicine, 450 Broadway Street, Pavilion B, 4th floor, Redwood City, CA 94063 USA; 3 Department of Dermatology, John H. Stroger Jr. Hospital of Cook County, Chicago, USA; 4 Department of Urology, Stanford University School of Medicine, Redwood City, USA; 5 Geriatrics Research Education and Clinical Center, Veterans Administration Health Care System, Palo Alto, USA; 6 Department of Medicine, Stanford University School of Medicine, San Francisco, USA; 7 Department of Dermatology, Emory University School of Medicine, Atlanta, USA

**Keywords:** Dermatology, Primary care, Geriatricians, Outpatient, Clinical care, Older adults, Geriatrics, Knowledge, Attitudes, Practices

## Abstract

**Background:**

According to the United States Census Bureau, the population aged ≥ 65 years is projected to increase, and the percentage of office visits from older adults to specialist physicians, such as dermatologists, will also increase. Despite older adults comprising an estimated 40% of dermatology visits currently, there is no formalized postgraduate curriculum on outpatient geriatric clinical care. Here, we performed an exploratory study to assess whether differences in the knowledge, attitudes and practices that are universal to outpatient geriatric clinical care exist among dermatologists compared to primary care or geriatric medicine, fields with formalized postgraduate curricula on outpatient geriatric clinical care.

**Methods:**

Following Institutional Review Boards’ approvals, a voluntary, anonymized online survey was conducted in 2023 of faculty physicians from Dermatology, Primary Care (Internal Medicine/ Family Medicine) and Geriatric Medicine departments/divisions at three academic centers, Stanford University, University of California San Francisco and Emory University. Validated survey items were used whenever possible. All statistical tests applied to the results were adjusted for multiple comparisons.

**Results:**

The overall response rate was 30.4% (146 completed surveys out of 480 invited). Knowledge of the 4Ms geriatric clinical framework was lowest amongst dermatologists (3.9%), followed by primary care physicians (17.8%) and geriatricians (100%). Attitudes on whether training program(s) provided adequate number of didactic lectures on clinical care of older adult patients indicated dermatologists responded with the lowest level of agreement (mean Likert score (MLS) 2.3, standard deviation (SD) 1.0) compared to primary care physicians’ responses (MLS 2.9, SD 1.1) and geriatricians (MLS 4.2, SD 1.4), p_adjusted_ < 0.05 for all comparisons. Practice differences in screening for mentation status were found, with dermatologists responding with lowest frequency (MLS 1.9, SD 1.2), followed by primary care physicians (MLS 3.7, SD 0.8), and geriatricians with highest frequency (MLS 4.5, SD 0.7), p_adjusted_ < 0.0001 for all comparisons.

**Conclusions:**

Our data suggests that differences exist in the knowledge, attitudes and practices of outpatient clinical care of older adults in specialty fields (in this case dermatology) compared to primary care and geriatric medicine, and are actionable findings. Additional investigation into whether these differences are found in other specialized fields could inform future postgraduate training and research.

**Supplementary Information:**

The online version contains supplementary material available at 10.1186/s12877-025-06628-8.

## Introduction

According to the United States Census Bureau, the population aged ≥ 65 years has grown by 38% from 2010–2020, is projected to continue to increase by 2030, and estimated to surpass the pediatric population by 2034 [1–3]. Over time, the percentage of office visits to specialist physicians of this age group have increased, and to primary care physicians have decreased [4]. However, whether differences exist in knowledge, attitudes and practices (KAPs) in outpatient geriatric clinical care by physicians in specialized fields compared to primary care or geriatric medicine is unclear. The KAPs that are universal to outpatient geriatric care have not been well-explored in specialty groups, and dermatology is one example.

The National Ambulatory Medical Care Survey 2015–2016 indicates that dermatologists have 44 million patient visits per year, with > 40% of their visits from patients ≥ 65 years or older [5]. Furthermore, patients aged ≥ 65 years have reported their preferred source of information on dermatologic concerns is dermatologists over primary care providers [6].

Despite this preference, familiarity of dermatologists with common geriatric frameworks and age-friendly practices is not clear. For instance, one of the most commonly utilized clinical frameworks in geriatric medicine is the 4Ms framework (comprising what matters, medications, mentation and mobility) which was developed in 2017 to improve reliability of evidence-based care to older adults [7–10]. Previous works have highlighted examples of the relevance of this framework for dermatologists, including goals of care, shared decision making, assessment of mentation for ability to consent for procedures, ability to adhere to treatment plans, avoidance of potentially inappropriate medications (ie. first generation antihistamines) or telemedicine for older patients with poor mobility, to name only a few examples [7–12]. Notably, dermatology does not currently have a formalized postgraduate training curriculum in geriatric clinical care, despite the many special medical considerations in older adults [7].

Here, we conduct a pilot, exploratory study to better understand whether response differences exist on KAPs of patients ≥ 65 years between dermatologists and primary care physicians, with geriatricians serving as the reference group, to serve as a starting point of where potential educational and research efforts could be targeted.

## Methods

### Survey design

The 19-item survey instrument is shown in Supplementary Fig. 1. It consisted of 17 content items on knowledge (6 items), attitudes (6 items), and practices (5 items) pertaining outpatient geriatric clinical care, and two additional exploratory questions: one on barriers and one on resources used in outpatient care of older adults.

Survey items and validity were developed by a team consisting of board-certified geriatricians with completion of internal medicine training (C.P., D.K., S.T.), a board-certified geriatric emergency medicine physician (W.P.), and board-certified dermatologists experienced in caring for older adults (A.S.C., K.A., H.Y.) [13]. Since no single available validated instrument in the literature encompassed the objectives of our study, our survey items were adapted from multiple publications after reviewing previous survey literature [7–9, 14–16]. To validate the questionnaire, the initial survey items were sent to 3 geriatricians and 3 medical dermatologists to evaluate the relevance of each question on a 4-point scale [13]. All finalized questions received an item-level content validity index (I-CVI) ≥ 0.83. All three subscales (knowledge, attitude, practice) received a scale-level content validity index based on the average method (S-CVI/Ave) ≥ 0.83.

Study data were collected and managed using Qualtrics XM Survey software hosted by Stanford and University of California, San Francisco (UCSF).

The study was carried out in accordance with the guidelines of the Declaration of Helsinki. Following approval of study procedures by the Emory University Institutional Review Board (IRB) (2022), Stanford University Human Subjects’ Panel (2023) and University of California San Francisco Human Research Protection Program’s IRB (2023), we conducted a voluntary, anonymized online survey at Stanford University, Emory University, and UCSF health care systems from April 12, 2023 to September 21, 2023. Survey invitations were emailed to faculty physician members with adult outpatient clinical practices in the departments of Dermatology, Primary Care (Internal Medicine or Family Medicine) and Geriatric Medicine (with Geriatric Medicine serving as a positive control or “gold standard”). Electronic informed consent was obtained from each participant prior to survey commencement. A gift certificate of $5 was sent to respondents who completed surveys. To maximize response rate, each potential participant received a total of two repeat emails. Inclusion criteria consisted of current practice involving outpatient care of older adults.

### Statistical analysis

Survey item responses in the “Attitudes” and “Practices” sections were scored on a Likert scale for level of agreement and coded from 1 to 5 points with assignment as follows: 1 = strongly disagree, 2 = somewhat disagree, 3 = neither agree nor disagree, 4 = somewhat agree, 5 = strongly agree. Survey item responses in the “Practices” section pertaining to frequency of practice were coded from 1 to 5 points with assignment as follows: 1 = never, 2 = sometimes, 3 = about half the time, 4 = most of the time, 5 = always.

The Wilcoxon rank-sum test or Fisher’s Exact test was applied, with significance threshold < 0.05 after adjustment for false discovery rate (Bonferroni correction). Analysis was performed on RStudio (Version 2023.03.0).

## Results

### Demographic characteristics of faculty physician respondents

The overall response rate was 30.4% (146/480 surveys returned), with 31.4% (61/194) response rate from UCSF physicians, 18.3% (34/186) response rate from Emory, and 51% (51/100) from Stanford. Response rates by physician group were: dermatologists 33.8% (26/77), primary care physicians 26.7% (90/337) and geriatricians 45.5% (30/66). Seven respondents were excluded from analysis as they reported current inpatient but no outpatient clinical care of older adults (these seven were all geriatricians).

Of the 139 respondents included in the analysis (26 dermatologists, 90 primary care physicians, 23 geriatricians), most physicians identified as female gender across all 3 physician groups (Table 1). The most common race was White for all 3 physician groups. Neither gender nor race were statistically significant across the 3 physician groups. The median years since last residency training and the median years since last fellowship training were not significantly different between the three physician groups.

**Table 1:** Demographic characteristics of survey respondents

Parameter		Dermatology *N* = 26	Primary Care *N* = 90	Geriatric Medicine *N* = 23	Total *N* = 139
		Number (%)	Number (%)	Number (%)	Number (%)
Sex	Female	21 (81)	64 (71)	20 (87)	105 (76)
	Male	5 (19)	23 (26)	3 (13)	31 (22)
	Prefer to not disclose	0 (0)	3(3)	0 (0)	3 (2)
Race	White	11 (42)	32 (36)	9 (39)	52 (37)
	Asian	5 (19)	29 (32)	8 (35)	42 (30)
	Native Hawaiian and other Pacific Islanders	0 (0)	1(1)	0 (0)	1 (1)
	Black or American African	1 (4)	6 (7)	0 (0)	7 (5)
	Hispanic	1 (4)	3 (3)	1 (4)	5 (4)
	Multi-ethnic	1 (4)	1 (1)	3 (13)	5 (4)
	Other	5 (19)	13 (14)	1(4)	19 (14)
	Prefer to not disclose	2 (8)	5 (6)	1 (4)	8 (6)
Median years since last residency training (range)^*^		8.7 (1.1—43.2)	12.9 (1.0—37.7)	14.2 (1.9—35.2)	12.2 (1.0—43.2)
Median years since last fellowship training (range)^*^		9.5 (3.2—41.3)	9.2 (0.9—28.8)	8.3 (0.2—25.2)	9.0 (0.2—41.3)

There were no significant differences in any of the parameters between dermatology, primary care and geriatric medicine, with all *p*-values > 0.05

^*^Wilcoxon rank-sum test (as data are not normally distributed)

Dermatologists and primary care physicians reported that about half of their outpatient patient population were older adults (mean percentage was 45% and 50%, respectively), while geriatricians reported that 100% of their patients were older adults.

### Knowledge responses on older adult clinical care

Responses to the knowledge-related survey item pertaining to familiarity with the 4Ms older adult care model were different between all 3 physician groups (Table 2). Only 3.9% of dermatologists responded “yes” to this item, compared to 17.8% of primary care physicians, and 100% of geriatricians [7, 8]. These differences were not significant between dermatologists’ versus primary care physicians’ responses (p_adjusted_ = 0.3426), but were significant between primary care physicians’ and geriatricians’ responses (p_adjusted_ < 0.0001). Dermatologists’ versus geriatricians’ responses were also significantly different (p_adjusted_ < 0.0001). There were no differences in responses among any of the 5 survey items on knowledge of older adult clinical care between dermatologists, primary care physicians and geriatricians.

**Table 2:** Survey responses to knowledge questions on outpatient clinical care of older adults among physician respondents

**Knowledge-Related Survey Item**	**Answer Choices**	**D** ***N* = 26**	**PC** ***N* = 90**	**G** ***N* = 23**	**D vs. PC** **P**_**adjusted**_	**D vs** **G** **P**_**adjusted**_	**PC vs. G** **P**_**adjusted**_
		Number (%) of “yes” responses					
Are you familiar with the 4Ms older adult care model? [7, 8]	Yes	1 (3.9)	16 (17.8)	23 (100)	0.3426	**< **0.0001	**< **0.0001
	No						
		Number (%) of correct responses					
When prescribing renally excreted medications, the serum creatinine level in older adults may not be an accurate reflection of renal function due to? [9]	Increased cholesterol	26 (100)	90 (100)	23 (100)	1	1	1
	Increased fat deposits						
	Loss of bone mass						
	Loss of muscle mass*						
Which of the following is a true statement? [16]	Chronological age is functional age	24 (92.3)	89 (98.9)	23 (100)	0.3771	1	1
	Functional age is a measure of the functional capacity of a person*						
	Functional age and chronological age increase at the same rate						
	Weight directly impacts chronological age						
A decline in mobility can impact older adults by? [9]	Decreasing ability to attend in-person clinic visits*	26 (100)	85 (94.4)	23 (100)	1	1	1
	Decreasing ability to make their own health care decisions						
	Decreasing need for home health assistance						
	Decreasing their concern about health						
Polypharmacy is associated with which of the following? [9]	Increased adherence	26 (100)	90 (100)	23 (100)	1	1	1
	Prescribing multiple medications with the same mechanism to treat the same condition is less likely to occur						
	Increased risk of drug-to-drug interaction*						
	Prescribing medications to treat side effects of other medications is less likely to occur						
Lag time to benefit is? [15]	Time from diagnosis to manifestation of the administered therapy’s benefit	26 (100)	83 (92.2)	21 (91.3)	1	0.6453	1
	Time from diagnosis to therapy administration						
	Time for intervention to manifest intended therapeutic benefit*						
	Time from intervention to death						

*P*-values adjusted for false discovery rate

*D* Dermatologists, *PC* Primary care physicians, *G* Geriatricians

^*^Indicates correct answer

### Attitudes on older adult clinical care

Differences across all 3 physician groups’ responses were found for the attitudes-related survey item “My training program(s) had an adequate number of didactic lectures on the clinical care of older adult patients.” Mean Likert score (MLS) [standard deviation (SD)] of agreement for this survey item was lowest for dermatologists at 2.3 [1.0], with increased scores by primary care physicians at 2.9 [1.1], and highest scores by geriatricians at 4.2 [1.4] (Table 3). These scores were significantly different between dermatologists’ versus primary care physicians’ responses (p_adjusted_ = 0.045), primary care physicians’ versus geriatricians’ responses (p_adjusted_ < 0.0001), and dermatologists’ versus geriatricians’ responses (p_adjusted_ < 0.0003).

**Table 3:** Survey responses to attitudes questions on outpatient clinical care of older adults among physician respondents

**Survey Item**	**D** Mean response (SD)	**PC** Mean response (SD)	**G** Mean response (SD)	**D vs. PC** **P**_**adjusted**_	**D vs. G** **P**_**adjusted**_	**PC vs. G** **P**_**adjusted**_
My training program(s) had an adequate number of didactic lectures on the clinical care of older adult patients	2.3 (1.0)	2.9 (1.1)	4.2 (1.4)	0.045	0.0003	<.0001
My training program(s) adequately trained me to provide high quality clinical care of older adult patients	3.3 (1.1)	3.2 (1.2)	4.3 (1.3)	1	0.003	<.0001
Topics related to older adults are well represented in my field of medicine's peer-reviewed literature	3.0 (1.0)	3.6 (1.0)	3.9 (1.4)	0.0585	0.0237	0.2259
Older adult topics are well represented at my field of medicine's national, regional, and institutional conference lectures/workshops	3.0 (1.0)	3.5 (1.0)	4.3 (1.1)	0.0519	0.0006	<.0001
If I had a question regarding a nuance of older adult care, my field of medicine would have adequate peer-reviewed journal articles to help answer this question	3.0 (1.0)	3.5 (1.0)	3.7 (1.3)	0.0729	0.0936	0.8466
In the past year, I have had at least one diagnostic dilemma in clinic regarding an older adult's care management due to the special clinical considerations in the older adult population	4.4 (0.8)	4.6 (0.6)	4.8 (0.4)	1	0.1584	0.1902

*P*-values adjusted for false discovery rate. *D* Dermatologists, *PC* Primary care physicians, *G* Geriatricians, *SD* Standard deviation. Survey item responses were scored on a Likert scale for level of agreement and were coded from 1 to 5 points with assignment as follows: 1 = strongly disagree, 2 = somewhat disagree, 3 = neither agree nor disagree, 4 = somewhat agree, 5 = strongly agree

Besides this survey item, none of the other survey items in the “attitudes” section demonstrated significantly different responses between dermatologists and primary care physicians.

Dermatologists’ responses to three other attitudes-related survey items were significantly different from the geriatrician’s responses, with dermatologists’ responses indicating less agreement with these statements than geriatricians’ responses. These three survey items were: “My training program adequately trained me to provide high quality clinical care of older adult patients” (p_adjusted_ = 0.003), “Topics related to older adults are represented in my field of medicine’s peer-reviewed literature” (p_adjusted_ = 0.0237), and “Older adult topics are well represented in my field of medicine’s national, regional and institution conference lectures/workshops” (p_adjusted_ = 0.0006).

Primary care physicians’ responses to these same three attitudes-related survey items were different from geriatricians’ responses in only two out of these three survey items, namely: “My training program adequately trained me to provide high quality clinical care of older adult” and “Older adult topics are well represented in my field of medicine’s national, regional and institution conference lectures/workshops”. Primary care physicians’ responses indicated less agreement by MLS than geriatricians’ responses to both these items, with p_adjusted_ < 0.0001 for both attitudes-related survey items.

### Practices in older adult clinical care

Significant differences were found between all 3 physician groups’ responses to the practices-related survey item “I screen for dementia, delirium, and/or depression in my older adult patients”, with dermatologists’ responses indicating mean response between “never” and “sometimes” (MLS = 1.9 [SD 1.2], primary care physicians’ responses indicating mean response between “about half the time” and “most of the time” (MLS = 3.7 [0.8]) and geriatricians’ responses mean response between “most of the time” and “always” (MLS = 4.5 [0.7]) (Table 4). These differences were statistically significant between dermatologists’ versus primary care physicians’ responses (p_adjusted_ < 0.0001), dermatologists’ versus geriatricians’ responses (p_adjusted_ < 0.0001), and primary care physicians’ versus geriatricians’ responses (p_adjusted_ < 0.0001).

**Table 4:** Survey responses to practices questions on outpatient clinical care of older adults among physician respondents

**Survey Item**	**D** Mean response (SD)	**PC** Mean response (SD)	**G** Mean response (SD)	**D vs. PC** **P**_**adjusted**_	**D vs. G** **P**_**adjusted**_	**PC vs. G** **P**_**adjusted**_
Prior to prescribing medications to older adults, I review their medication list	4.5 (0.8)	4.8 (0.5)	5.0 (0.2)	0.0783	0.0201	0.3279
If the nature of the visit allows and I have the resources, I would offer telemedicine visits for older adults with limited mobility	4.7 (0.8)	4.8 (0.5)	5.0 (0.2)	1	0.3591	0.3345
I use patient decision aids (video, pamphlets, images/photos, etc.) to discuss medications and procedural therapies with older adult patients	3.0 (1.4)	2.8 (1.1)	3.0 (1.0)	1	1	1
I screen for dementia, delirium, and/or depression in my older adult patients	1.9 (1.2)	3.7 (0.8)	4.5 (0.7)	< 0.0001	< 0.0001	< 0.0001
I read articles on topics regarding older adult clinical care models in my field of medicine's peer-reviewed literature	2.2 (0.9)	2.7 (1.0)	4.5 (0.9)	0.1242	< 0.0001	< 0.0001

*P*-values adjusted for false discovery rate. *D* Dermatologists, *PC* Primary care physicians, *G* Geriatricians, *SD* Standard deviation. Survey item responses were scored on a Likert scale for level of agreement and coded from 1 to 5 points with assignment as follows: 1 = strongly disagree, 2 = somewhat disagree, 3 = neither agree nor disagree, 4 = somewhat agree, 5 = strongly agree. Some survey item responses in this section queried frequency of practice, and were coded from 1 to 5 points with assignment as follows: 1 = never, 2 = sometimes, 3 = about half the time, 4 = most of the time, 5 = always

Other differences in practice-related survey items included a lower MLS for dermatologists compared to geriatricians (4.5 [SD 0.8] versus 5.0 [0.2] respectively, p_adjusted_ = 0.0201) on the statement “Prior to prescribing medications to older adults, I review their medication list.” The MLS for this item was also lower for dermatologists’ responses compared to primary care physicians (4.5 [SD 0.8] versus 4.8 [0.5] respectively which showed a trend toward significance, p_adjusted_ = 0.0783).

Finally, dermatologists’ responses indicated a lower MLS (2.2, [SD 0.9]) than primary care physicians’ (2.7 [1.0]) and geriatricians’ responses (4.5 [0.9]) to the practices survey item “I read articles on topical regarding older adult clinical care models in my field of medicine’s peer reviewed literature”. Only the scores between dermatologists’ versus geriatricians (p_adjusted_ < 0.0001) and primary care physicians versus geriatricians (p_adjusted_ < 0.0001) were significant though.

The survey item responses on attitudes and practices are shown as proportions in Table 5.

**Table 5:** Responses to survey items on attitudes and practices among dermatologists, primary care physicians and geriatricians. Results are shown as proportions

Questions on Attitudes	Specialty	Answer choices				
		**Strongly disagree**	**Somewhat disagree**	**Neither agree nor disagree**	**Somewhat agree**	**Strongly agree**
Adequate number of didactics on care of older adult patients?	D (%)	5 (19.2)	11 (42.3)	8 (30.8)	1 (3.8)	1 (3.8)
	PC (%)	9 (10)	30 (33.3)	14 (15.6)	32 (35.6)	5 (5.6)
	G (%)	2 (8.7)	2 (8.7)	1 (4.3)	3 (13)	15 (65.2)
Program trained on high quality clinical care of older adult patients?	D (%)	1 (3.8)	5 (19.2)	7 (26.9)	10 (38.5)	3 (11.5)
	PC (%)	5 (5.6)	25 (27.8)	15 (16.7)	33 (36.7)	12 (13.3)
	G (%)	2 (8.7)	1 (4.3)	1 (4.3)	2 (8.7)	17 (73.9)
Topics on older adults represented in field’s literature?	D (%)	1 (3.8)	8 (30.8)	7 (26.9)	9 (34.6)	1 (3.8)
	PC (%)	2 (2.2)	17 (18.9)	13 (14.4)	44 (48.9)	14 (15.6)
	G (%)	3 (13)	1 (4.3)	1 (4.3)	9 (39.1)	9 (39.1)
Older adult topics represented in field’s conference lectures/workshops?	D (%)	0	11 (42.3)	7 (26.9)	6 (23.1)	2 (7.7)
	PC (%)	2 (2.2)	16 (17.8)	19 (21.1)	43 (47.8)	10 (11.1)
	G (%)	1 (4.3)	1 (4.3)	2 (8.7)	4 (17.3)	15 (65.2)
Adequate peer-reviewed journal articles for answering questions on older adult care?	D (%)	2 (7.7)	6 (23.1)	9 (34.6)	8 (30.8)	1 (3.8)
	PC (%)	3 (3.3)	10 (11.1)	27 (30)	37 (41.1)	13 (14.4)
	G (%)	2 (8.7)	4 (17.4)	0	11 (47.8)	6 (26.1)
> 1 diagnostic dilemma regarding older adult care in the past year?	D (%)	0	1 (3.8)	2 (7.7)	8 (30.8)	15 (57.7)
	PC (%)	0	0	3 (3.3)	31 (34.4)	56 (62.2)
	G (%)	0	0	0	4 (17.4)	19 (82.6)
**Questions on Practices**	**Specialty**	**Strongly disagree**	**Somewhat disagree**	**Neither agree nor disagree**	**Somewhat agree**	**Strongly agree**
Offer telemedicine visits for older adults with limited mobility?	D (%)	0	2 (7.7)	0	3 (11.5)	21 (80.8)
	PC (%)	0	0	2 (2.2)	14 (15.6)	74 (82.2)
	G (%)	0	0	0	1 (4.3)	22 (95.7)
		**Never**	**Sometimes**	**About half the time**	**Most of the time**	**Always**
Medication list review prior to prescribing medications to older adults?	D (%)	0	1 (38.5)	2 (76.9)	7 (26.9)	16 (61.5)
	PC (%)	0	0	4 (4.4)	12 (13.3)	74 (82.2)
	G (%)	0	0	0	1 (4.3)	22 (95.7)
Use patient decisions aids to discuss with older adult patients?	D (%)	3 (11.5)	9 (34.6)	3 (11.5)	6 (23.1)	5 (19.2)
	PC (%)	7 (7.8)	41 (45.6)	10 (11.1)	27 (30)	5 (5.6)
	G (%)	0	10 (43.5)	5 (21.7)	7 (30.4)	1 (4.3)
Screen for dementia, delirium, and/or depression?	D (%)	13 (50)	8 (30.8)	1 (3.8)	3 (11.5)	1 (3.8)
	PC (%)	0	12 (13.3)	12 (13.3)	55 (61.1)	11 (12.2)
	G (%)	0	1 (4.3)	0	8 (34.8)	14 (60.9)
Read articles on older adult clinical care models in field’s literature?	D (%)	4 (15.4)	15 (57.7)	5 (19.2)	1 (3.8)	1 (3.8)
	PC (%)	5 (5.6)	45 (50)	18 (20)	18 (20)	4 (4.4)
	G (%)	0	2 (8.7)	1 (4.3)	3 (13)	17 (73.9)

Question wording is abbreviated in this table, however, complete wording as presented to the responders is available in Supplemental Fig. 1

*D* Dermatologists, *PC* Primary care physicians, *G* Geriatricians

### Additional exploratory survey item on resources used when a question arises regarding an older adult patient

In response to the survey item, “If I have a question about a topic regarding one of my older adult patients, I would refer to:”, there were no differences in resources used between dermatologists’ and primary care physicians’ responses except for the resource of “peer reviewed journals in my field” (88.5% versus 58.9% respectively; p_adjusted_ = 0.0147) (Table 6). There were no differences in resources used between dermatologists’ and geriatricians’ responses. Primary care physicians’ response to using “peer reviewed journals in my field” were lower than geriatrician’s response (58.9% versus 87.0%, respectively; p_adjusted_ = 0.042). Primary care physicians’ response to “refer to a colleague within my field” was lower than geriatricians’ response (44.4% versus 78.3%, respectively; p_adjusted_ = 0.0141).

**Table 6:** Survey responses to resources utilized in outpatient clinical care of older adults among physician respondents

**Response**	**D** Number (%)	**PC** Number (%)	**G** Number (%)	**D vs. PC** **P**_**adjusted**_	**D vs. G** **P**_**adjusted**_	**PC vs. G** **P**_**adjusted**_
Peer reviewed journals in my field	23 (88.5)	53 (58.9)	20 (87.0)	0.0147	1	0.042
Peer reviewed journals outside my field	15 (57.7)	28 (31.1)	13 (56.5)	0.0612	1	0.09
Non-peer reviewed journals	0 (0.0)	2 (2.2)	2 (8.7)	1	0.6453	0.5505
Primary internet source (UpToDate or WebMD)	16 (61.5)	76 (84.4)	20 (87.0)	0.0744	0.1719	1
Social media	0 (0)	0 (0)	1 (4.3)	1	1	0.6105
Refer to a colleague within my field	13 (50.0)	40 (44.4)	18 (78.3)	1	0.2208	0.0141
Refer to a colleague outside in field	8 (30.8)	47 (52.2)	9 (39.1)	0.2223	1	1
Other	0 (0)	8 (8.9)	1 (4.3)	0.5877	1	1

The survey item was “If I have a question about a topical regarding one of my older adult patients, I would refer to (check all that apply)”, with responses shown in the table above. *P*-values calculated with Fisher’s exact test and adjusted for false discovery rate

*D* Dermatologists, *PC* Primary care physicians, *G* Geriatricians

### Additional exploratory survey item on barriers to implementing older adult clinical care models in practice

In general, responses to the survey item “Barriers to implementing older adult clinical care models into my practice are:” were similar between dermatologists’ and primary care physicians’ responses, except that more dermatologists’ responses indicated “limited studies in the literature on the utility of older adult care models” as a barrier (46.2%) compared to primary care physicians’ responses (15.6%), p_adjusted_ = 0.0072 (Table 7). A higher percentage of geriatricians’ responses than primary care physicians’ responses indicated the barrier of “limited studies in the literature on the utility of older adult clinical care models” (47.8% versus 15.6% respectively, p_adjusted_ = 0.0102). Notably, this pattern is consistent with the survey item on resources used (see section above), in which both dermatologists and geriatricians had a higher percentage utilizing peer reviewed journals in their fields as a resource, compared to primary care physicians (Table 6).

**Table 7:** Survey responses to perceived barriers to implementing older adult clinical care models into practice among physician respondents

**Response**	**D** Number (%)	**PC** Number (%)	**G** Number (%)	**D vs. PC** **P**_**adjusted**_	**D vs. G** **P**_**adjusted**_	**PC vs. G** **P**_**adjusted**_
My limited knowledge of older adult clinical care models	15 (57.7)	39 (43.3)	1 (4.3)	0.7941	< 0.0001	0.0012
Limited time in the clinic setting	22 (84.6)	84 (93.3)	20 (87.0)	0.6837	1	1
Limited studies in the literature on the utility of older adult care models	12 (46.2)	14 (15.6)	11 (47.8)	0.0072	1	0.0102
Lack of general interest in my field to advocate for older adults' specific issues	1 (3.8)	5 (5.6)	5 (21.7)	1	0.2586	0.0855
Lack of interest in my field to implement systemic change	3 (11.5)	15 (16.7)	5 (21.7)	1	1	1
Lack of recognition that older adults are a vulnerable population	4 (15.4)	8 (8.9)	8 (34.8)	1	0.5505	0.012
Older adult clinical care models are outside the scope of my practice	2 (7.7)	2 (2.2)	2 (8.7)	0.6498	1	0.5505

The survey item was: “Barriers to implementing older adult clinical care models into my practice are:”, with responses shown in the table above. *P*-values calculated with Fisher’s exact test and adjusted for false discovery rate

*D* Dermatologists, *PC* Primary care physicians, *G* Geriatricians

Both dermatologists’ and primary care physicians’ responses had a higher percentage selecting “My limited knowledge of older adult clinical care models” as a barrier (57.7% and 43.3% respectively) compared to geriatricians’ responses (4.3%), with p_adjusted_ < 0.0001 for dermatologists’ versus geriatricians’ responses, and p_adjusted_ = 0.0012 for primary care physicians’ versus geriatricians’ responses to this answer choice, as expected.

More geriatricians responded that “lack of recognition that older adults are a vulnerable population” was a barrier (34.8%) than dermatologists (15.4%) and primary care physicians (8.9%). Among the 3 physician groups, only the difference between geriatricians’ and primary care physicians’ responses were significantly different (p_adjusted_ = 0.012).

## Discussion

The American Association of Medical Colleges (AAMC) has projected a 20% increase in patient demand for medical specialties (which includes dermatology) from 2014 to 2025, in large part due to the aging population [2–4]. Demand for outpatient clinical care of older of older adults in dermatology has been predicted to grow at an even higher rate due to increasing incidences of skin cancer and growing interest in aesthetic [17].

Our data is an initial step that has identified feasible and actionable steps to potentially reduce the differences between dermatologists and primary care physicians with those who are experts at older adult care, the geriatricians. Differences in knowledge on geriatric clinical care frameworks could be addressed by implementing a curriculum in dermatology that incorporates these steps. Our dermatologists’ responses indicate that they recognize their training programs may not have an adequate number of didactic lectures on the clinical care of older adult patients, and hence, the “buy-in” for incorporating geriatric clinical care frameworks may already be present within the field. Note that although didactic lectures may not capture all modalities of training during residency, it remains the main form of teaching during this phase of training [18], and is therefore one suitable indicator for the level of geriatric clinical care education.

The practice difference that dermatologists screen for dementia, delirium, and/or depression in older adult patients less frequently than primary care and geriatricians is especially important in light of shared decision-making in older adults to optimize clinical outcomes [19]. Mentation status determines whether an older adult patient can weigh the pros and cons of treatment and therefore consent for treatments. Dementia, delirium and depression can certainly negatively impact an older patient’s decision-making capacity, but having one of these mentioned conditions does not automatically translate into the lack of capacity [10]. Instead, assessment by the dermatologist of the level to which dementia, delirium or depression are impacting the four decision-making abilities (understanding, expressing a choice, appreciation, and reasoning) is needed [10]. Furthermore, mentation status is critical for treatment adherence, and may flag a patient in need of caregiver assistance. Common dermatologic medications such as antihistamines, acyclovir and benzodiapines (often used prior to procedures) can contribute to delirium [9]. Depression is associated with common skin diseases such as atopic dermatitis and psoriasis, and is often screened for with validated questionnaires, such as PHQ-2, by dermatologists. It is very treatable and when recognized, appropriate referrals can be made for optimal care. When severe, depression can impact decision-making capacity and/or adherence to treatment plans [20]. Time efficient and effective mentation screening tools feasible for use in outpatient clinic settings could be reviewed as part of standard dermatology postgraduate curriculum to reduce practice differences between dermatology, primary care, and geriatrics.

Regarding the lower likelihood of reviewing medication lists by dermatologists prior to prescribing medications, our survey item does not specify if the prescribed medication is topical or systemic. The lower rates of reviewing medication lists could be due to lack of checking when prescribing topical agents unlikely to interact with other medications. However, reviewing medications for interactions is critical to optimal patient care, as systemic medications (e.g. biologic agents or targeted immunosuppressive drugs) are more frequently being used by dermatologists [21, 22].

Additional studies may shed light on whether other specialty groups with comparable rates of outpatient older adult populations (such as ophthalmology, psychiatry or otolaryngology) demonstrate similar patterns to dermatology, compared to primary care medicine or geriatrics on the outpatient clinical care of older adults.

The major limitation of our study is the overall response rate, which may lead to biased results in favor of faculty physicians who have more interest in outpatient clinical care of older adults. Nevertheless, we believe that our results are still worth considering, as this type of data has not been previously reported, and this is an exploratory study whose results may spark additional larger investigation, with increased numbers of academic institutions as well as community physicians.

## Conclusions

Our data identified differences in knowledge of a common geriatric clinical care model of 4Ms in both dermatologists and primary care physicians. Regarding attitudinal differences, we found dermatologists were less likely to indicate that their training programs had an adequate number of didactic lectures on the clinical care of older adult patients, which is consistent with the lack of formal postgraduate curriculum in geriatric dermatology. We also identified that dermatologists screen for dementia, delirium, and/or depression in older adult patients less frequently than primary care and geriatricians. This practice difference is especially important in light of the push toward shared decision-making in older adults to optimize clinical outcomes, as the mentation status of an older patient underlies their ability to weigh the pros and cons of treatment and consent for treatments [19].

## Supplementary Information

Supplementary Material 1.

## Data Availability

All data supporting the findings of this study are available within the paper. If additional information is needed, please contact the corresponding author and data will be made available upon reasonable request.

## Abbreviations

I-CVI: Item-level content validity index
KAP: Knowledge, attitudes and practices
MLS: Mean likert scale
S-CVI: Scale-level content validity index
SD: Standard deviation
UCSF: University of California at San Francisco
