# Supplementary material for: Exploratory survey study of differences in knowledge, attitudes, and practices of outpatient older adult clinical care between dermatologists, primary care physicians and geriatricians across three academic medical centers
Source: BMC Geriatr. 2026 Jan 29;26:256. doi: 10.1186/s12877-025-06628-8 (PMC12924514; doi:10.1186/s12877-025-06628-8)
Supplement: Supplementary file 1 — Supplementary Material 1. [file 12877_2025_6628_MOESM1_ESM.docx]

Knowledge

Q: Are you familiar with the 4Ms older adult care model?

- No
- Yes

Q: When prescribing renally excreted medications, the serum creatinine level in older adults may not be an accurate reflection of renal function due to:

- Increased cholesterol
- Increased fat deposits
- Loss of bone mass
- Loss of muscle mass

Q: Which of the following is a true statement?

- Chronological age is synonymous with functional age.
- Functional age is a measure of the functional capacity of a person.
- Functional age and chronological age increase at the same rate.
- Weight directly impacts chronological age.

Q: A decline in mobility can impact older adults by:

- Decreasing the ability to attend in-person clinic visits.
- Decreasing the ability to make their own health care decisions.
- Decreasing their need for home health assistance.
- Decreasing their concern about their health.

Q: Polypharmacy is associated with which of the following?

- Increased adherence due to increased number of medications
- Prescribing multiple medications with the same mechanism of action to treat the same condition is less likely to occur
- Increased risk of drug-to-drug interaction
- Prescribing medications to treat side effects of other medications is less likely to occur

Q: Lag time to benefit is:

- Time from diagnosis to the manifestation of the administered therapy’s benefit.
- The time from diagnosis to the administration of therapy.
- The time it takes for an intervention to manifest its intended therapeutic benefit after being performed/administered.
- The time from intervention to death of patient.

Attitudes

Q: My training program(s) had an adequate number of didactic lectures on the clinical care of older adult patients.

- Strongly disagree
- Somewhat disagree
- Neither agree nor disagree
- Somewhat agree
- Strongly agree

Q: My training program(s) adequately trained me to provide high quality clinical care of older adult patients.

- Strongly disagree
- Somewhat disagree
- Neither agree nor disagree
- Somewhat agree
- Strongly agree

Q: Topics related to older adults are well represented in my field of medicine’s peer-reviewed literature.

- Strongly disagree
- Somewhat disagree
- Neither agree nor disagree
- Somewhat agree
- Strongly agree

Q: Older adult topics are well represented at my field of medicine’s national, regional, and institutional conference lectures/workshops.

- Strongly disagree
- Somewhat disagree
- Neither agree nor disagree
- Somewhat agree
- Strongly agree

Q: If I had a question regarding a nuance of older adult care, my field of medicine would have adequate peer-review journal articles to help answer this question.

- Strongly disagree
- Somewhat disagree
- Neither agree nor disagree
- Somewhat agree
- Strongly agree

Q: In the past year, I have had at least one diagnostic dilemma in clinic regarding an older adult’s care management due to the special clinical considerations in the older adult population.

- Strongly disagree
- Somewhat disagree
- Neither agree nor disagree
- Somewhat agree
- Strongly agree

**Practices**

Q: Prior to prescribing medications to older adults, I review their medication list.

- Never
- Sometimes
- About half the time
- Most of the time
- Always

Q: If the nature of the visit allows and I have the resources, I would offer telemedicine visits for older adults with limited mobility.

- Strongly disagree
- Somewhat disagree
- Neither agree nor disagree
- Somewhat agree
- Strongly agree

Q: I use patient decision aids (videos, pamphlets, images/photos, etc.) to discuss medications and procedural therapies with older adult patients.

- Never
- Sometimes
- About half the time
- Most of the time
- Always

Q: I screen for dementia, delirium, and/or depression in my older adult patients.

- Never
- Sometimes
- About half the time
- Most of the time
- Always

Q: I read articles on topics regarding older adult clinical care models in my field of medicine’s peer-reviewed literature.

- Never
- Sometimes
- About half the time
- Most of the time
- Always

**Additional Questions**

Q: If I have a question about a topic regarding one of my older adult patients, I would refer to (Check all that apply):

- Peer reviewed journals within my field
- Peer reviewed journals outside of my field
- Non-peer reviewed journals
- Primary internet source (such as Up-to-Date or WebMD)
- Social media
- Refer to a colleague within my field
- Refer to a colleague outside of my field
- Other: __________________________________________________

Q: Barriers to implementing older adult clinical care models into my practice are (Check all that apply):

- My limited knowledge of older adult clinical care models
- Limited time in the clinic setting
- Limited studies in the literature on the utility of older adult care models
- Lack of general interest in my field to advocate for older adults' specific issues
- Lack of interest in my field to implement systemic change
- Lack of recognition that older adults are a vulnerable population
- Older adult clinical care models are outside the scope of my practice
